# Mechanisms of Disease Progression and Resistance to Tyrosine Kinase Inhibitor Therapy in Chronic Myeloid Leukemia: An Update

**DOI:** 10.3390/ijms20246141

**Published:** 2019-12-05

**Authors:** Luana Bavaro, Margherita Martelli, Michele Cavo, Simona Soverini

**Affiliations:** Department of Experimental, Diagnostic and Specialty Medicine, Hematology/Oncology “L. e A. Seràgnoli”, University of Bologna, 40138 Bologna, Italy; luana.bavaro@studio.unibo.it (L.B.); margherita.martelli2@studio.unibo.it (M.M.); michele.cavo@unibo.it (M.C.)

**Keywords:** chronic myeloid leukemia, blastic phase, BCR-ABL1, tyrosine kinase inhibitors, resistance, persistence

## Abstract

Chronic myeloid leukemia (CML) is characterized by the presence of the *BCR-ABL1* fusion gene, which encodes a constitutive active tyrosine kinase considered to be the pathogenic driver capable of initiating and maintaining the disease. Despite the remarkable efficacy of tyrosine kinase inhibitors (TKIs) targeting BCR-ABL1, some patients may not respond (primary resistance) or may relapse after an initial response (secondary resistance). In a small proportion of cases, development of resistance is accompanied or shortly followed by progression from chronic to blastic phase (BP), characterized by a dismal prognosis. Evolution from CP into BP is a multifactorial and probably multistep phenomenon. Increase in BCR-ABL1 transcript levels is thought to promote the onset of secondary chromosomal or genetic defects, induce differentiation arrest, perturb RNA transcription, editing and translation that together with epigenetic and metabolic changes may ultimately lead to the expansion of highly proliferating, differentiation-arrested malignant cells. A multitude of studies over the past two decades have investigated the mechanisms underlying the closely intertwined phenomena of drug resistance and disease progression. Here, we provide an update on what is currently known on the mechanisms underlying progression and present the latest acquisitions on BCR-ABL1-independent resistance and leukemia stem cell persistence.

## 1. Introduction

Chronic Myeloid Leukemia (CML) is a clonal myeloproliferative disorder arising from a multipotent hematopoietic stem cell (HSC). CML is characterized by a unique cytogenetic abnormality involving the long arms of chromosomes 9 and 22, the t(9;22)(q34;q11) translocation, resulting in a derivative 22q- traditionally known as the Philadelphia chromosome [1]. The translocation juxtaposes the c-*ABL* oncogene 1 (*ABL1*) gene on chromosome 9 with the breakpoint cluster region (*BCR*) gene on chromosome 22, causing the formation of the *BCR-ABL1* fusion gene which encodes an oncoprotein with enhanced tyrosine kinase activity. It is believed that the acquisition of this fusion gene occurs in a single HSC that gains a proliferative advantage and/or aberrant differentiation capacity with consequent expansion of the myeloid compartment [2]. CML is characterized by a triphasic clinical course. More than 90% of CML cases are diagnosed in an early phase known as chronic phase (CP). Up to 50% of patients are asymptomatic at this stage and the disease is diagnosed by routine blood tests. Common findings at the time of diagnosis are fatigue, weight loss, abdominal fullness, bleeding, purpura, splenomegaly, leukocytosis, anemia, and thrombocytosis [1]. In this initial stage, mature granulocytes are still produced but there is an increased pool of myeloid progenitor cells in the peripheral blood (PB). If not effectively treated, CML may progress to an accelerated phase (AP), that is variable in duration and may be followed by a blastic phase (BP), which resembles morphologically acute leukemia: myeloid and/or lymphoid differentiation stops and immature blasts accumulate in the bone marrow (BM) spreading then to tissues and organs. Twenty to 25% of patients may develop BP without going through the intermediate AP. There is currently no consensus between the European Leukemia Net (ELN) and the World Health Organization (WHO) as to the percentage of blasts concurring to the definition of BP. According to the former, BP is defined by ≥30% blasts in the bone marrow or peripheral blood or extramedullary disease apart from spleen. The WHO instead sets the percentage of blasts required for BP definition at 20% [3]. Before the advent of *ABL1* tyrosine kinase inhibitors (TKIs), blasts were usually of myeloid phenotype in approximately 70% of CML-BP patients and of B-Lymphoid phenotype in approximately 30% of patients; rarely, mixed phenotypes were found. In the TKI-era, the blastic transformation of CML with mixed phenotype is getting even more rare. In the myeloid phenotype, blasts can be neutrophilic, eosinophilic, basophilic, monocytic, megakaryocytic, or erythroid [4,5,6]. Rarely, cases of T-lymphoid and promyelocytic-BP have been documented [7,8] and they are even more rare during TKI treatment [9,10]. Looking at gene expression profiling during the evolution, the progression from CP to advanced phases has been described as a two-step rather than a three-step process. Gene expression patterns resulted to be very similar between accelerated and blast phases and crucial steps in progression show up at the transition of CP to AP when a block of differentiation and apoptosis, alterations in cell adhesion, activation of alternative signaling pathways, and a shift toward turning on expression of genes involved in the nucleosome are observed [11].

CP CML patients can successfully be treated with *ABL1* TKIs. The first one to be introduced was imatinib mesylate, which showed a high rate of responses and an acceptable side effect profile when evaluated as initial therapy for newly diagnosed CP CML in the pivotal IRIS study. However, some patients may not respond to imatinib (primary resistance) and others relapse after an initial response (secondary resistance). In a small proportion of cases, development of resistance is accompanied or shortly followed by progression to BP. The problem of resistance has been the main trigger for the development of second-generation TKIs (2G-TKIs)―first approved for imatinib-resistant patients and later as frontline therapy. Today three 2G-TKIs (bosutinib, dasatinib, and nilotinib) are available and significant clinical benefit as compared to imatinib has been demonstrated by randomized clinical trials [12,13,14]. A third generation TKI (ponatinib) is also available for patients with a peculiar TKI-resistant mutation in the *BCR-ABL1* gene (the T315I, against whom imatinib and 2G-TKIs are ineffective; discussed in detail below) or for whom no other TKI is indicated. However, TKIs have only modestly improved survival of BP patients. Indeed, in a cohort study of 477 BP-CML patients, Jain et al. observed an overall survival (OS) of 12 months [15]. Therefore, preventing rather than counteracting disease progression is essential in an attempt to improve outcomes as well as healthcare resource utilization [15]. For almost two decades, a multitude of research studies have investigated the mechanisms underlying the closely intertwined phenomena of drug resistance and disease progression, with the aim to identify dysregulated molecules, validate new druggable targets and optimize treatment decision algorithms. This review provides a comprehensive overview of what is currently known and discusses what would deserve further investigation in an attempt to optimize patient outcomes.

## 2. Overview of Known Mechanisms Underlying Progression to BP

The *BCR-ABL1* oncogene arises in a primitive cell named leukemia stem cell (LSC), not yet committed to either myeloid or lymphoid differentiation. The blastic clone may originate either at the level of the multipotent LSC (CD34+CD38-Lin-) or at the level of a more committed granulocyte-macrophage progenitor cell, where the activation of β-catenin [16,17] and of hairy enhancer of split 1 (Hes1) [18] appear to be among the key events responsible for the re-acquisition of self-renewal capacity and enhancement of leukemic potential.

**Figure 1:**
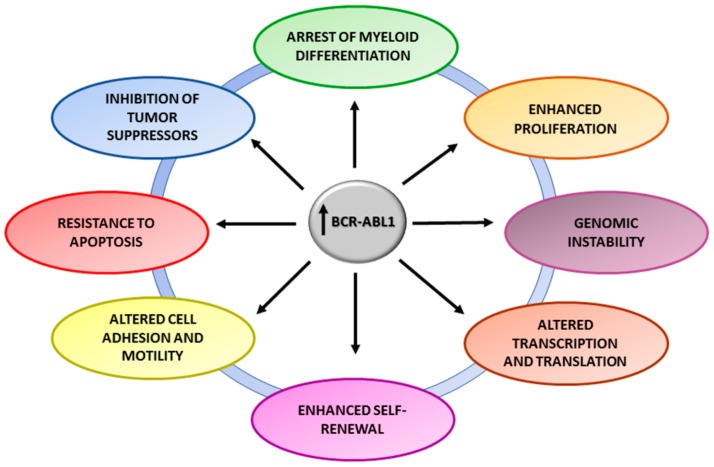
Overview of BCR-ABL1-dependent mechanisms. High BCR-ABL1 expression and activity in chronic myeloid leukemia (CML) implicate alteration of the normal cellular and genetic characteristics leading to transformation and progression to advanced phases.

Since the BCR-ABL1 oncoprotein is found in the cytoplasm [19], it can interact with many cellular substrates leading to abnormal pathways activation. By now, the oncoprotein is recognized to enhance proliferation activating Ras-mitogen-activated protein kinase (MAPK), to increase the transcriptional activity perturbing the JAK-STAT pathway, to decrease the apoptosis activating phosphatidylinositol 3-kinase (PI3K)/AKT pathway and to alter the expression of several genes/molecules implicated in cell adhesion and motility like L-selectin, *ICAM-1*, and *CCR7*. BCR-ABL1 is necessary for malignant transformation but not sufficient to sustain BP. As matter of fact, the expression levels of BCR-ABL1 (both at the mRNA and protein level) increase with disease progression [20,21], promoting the onset of secondary molecular and chromosomal “hits” which ultimately lead to the expansion of highly proliferating differentiation-arrested malignant cell clones. Once these ‘’hits’’ have been acquired, inhibiting BCR-ABL1 alone often fails, as testified by the low efficacy of TKI treatment in the advanced phases of the disease. However, although BCR-ABL1 is likely to be the primum movens responsible for the acquisition of many of the additional hits that have been implicated in disease progression, the fact that some patients suddenly progress while on TKI treatment suggests that disease progression may also involve BCR-ABL1-independent mechanisms. Evolution from CP into BP is a multifactorial and probably multistep phenomenon, and although hundreds of studies have been performed to dissect progression mechanisms, some pieces of the puzzle are still missing. It is believed that disease progression may be triggered by a series of distinct but functionally equivalent events in different patients, whereby the increase in BCR-ABL1 transcript levels cooperate with BCR-ABL1-dependent and –independent genomic instability to determine the accumulation of key events at the DNA, RNA and protein level that converge to subvert cell cycle control, differentiation, apoptosis (Figure 1).

## 3. BCR-ABL1 and Genomic Instability

The frequency of numerical and structural chromosomal changes results to be much higher in BP-CML compared with CP-CML. Studies in pediatric and adult CML patients showed that copy number alterations (CNAs) are rare or absent in CML-CP but increase upon disease progression, a sign of the increasing genomic instability [22,23,24,25,26,27].

**Figure 2:**
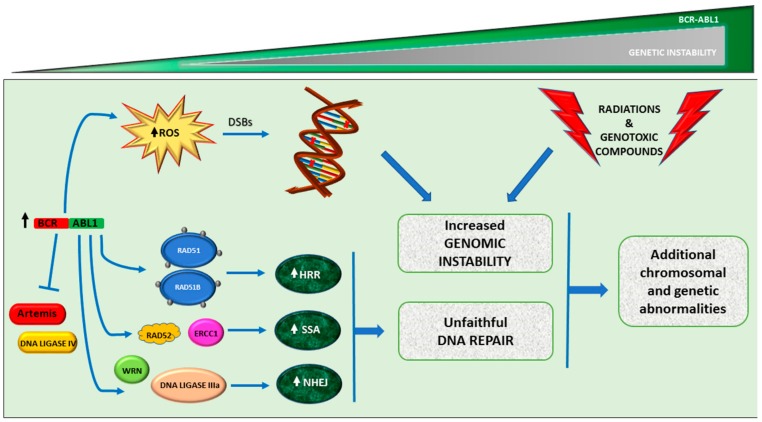
The role of BCR-ABL1 in the genomic instability. High levels of BCR-ABL1 are responsible for the generation of reactive oxygen species (ROS) and stimulate unfaithful DNA repair mechanisms (HRR= homologous recombination repair, NHEJ = nonhomologous end-joining, SSA = single-cell annealing) thus leading to increased DNA damage.

BCR-ABL1 is considered a direct cause of genomic instability since its expression and activity are responsible for the generation of reactive oxygen species (ROS), disruption of DNA repair pathways and inhibition of DNA damage-induced apoptosis that may result in aneuploidy, chromosomal alterations, DNA deletions and insertions, and point mutations (Figure 2). The involvement of ROS in genomic instability and CML progression has been extensively reviewed [28]. Controlling the levels of intracellular ROS is one of the mechanisms required to guarantee genomic integrity, because ROS can damage both the nucleobases and the deoxyribose incorporated in DNA as well as the free nucleotides, leading to oxidized bases and DNA double-strand breaks (DSBs). BCR-ABL1 kinase activity has been found to elevate intracellular ROS levels [29], and this was markedly more evident in BP CML cells, exhibiting higher BCR-ABL1 levels, than in CP CML cells [30]. If, on one hand, ROS levels and exogenous factors such as radiations or genotoxic compounds may enhance oxidative DNA damage, on the other hand the DNA repair machinery are deregulated by loss or gain of function in BCR-ABL1-positive cells [31]. In human cells, DSBs are preferentially repaired by homologous recombination (HR) or non-homologous end joining (NHEJ), but sometimes the highly unfaithful single-strand annealing (SSA) mechanism may operate [32]. Novicki et al. have demonstrated an enhanced activity of HR and NHEJ in the repair of ROS-mediated DSBs in BCR-ABL1 cells, where these mechanisms resulted to be unfaithful, causing mutations and large deletions. As matter of fact, it has later been demonstrated that BCR-ABL1 (non mutated and T315I mutant) may bind and phosphorylate RAD51, as well as its paralog, RAD51B, promoting unfaithful homologous HR in a dosage-dependent manner.

Another key player in HR, BRCA1, has been found to be downregulated in cell lines and primary samples from patients with CP and BP [33]. Reduced BRCA1 protein levels in CML has been ascribed to BCR-ABL1-dependent inhibition of BRCA1 mRNA translation [34] and to BCR-ABL1-dependent downregulation of the BRCA1-associated protein 1 (BAP1) deubiquitinase [35].

Since 2002 an increased error-prone repair by NHEJ had been remarked in myeloid leukemia cells. The ‘’canonical’’ NHEJ pathway in human cells is triggered by the recognition and binding of DSBs by Ku70 and Ku80, which recruit the DNA-dependent protein kinase catalytic subunit (DNA-PKcs) to form the functional DNA-dependent protein kinase (DNA-PK). DNA-PK serves as a bridge to mediate the ligation of DNA ends by the DNA ligase IV (LIG4) complex and activates the Artemis nuclease which is required, together with DNA polymerases mu and lambda (Pol μ and Pol λ,), to create ligatable ends [36]. However, an alternative (‘‘backup’’) NHEJ, slow and error-prone, may be carried out by a different subset of proteins―including poly (ADP-ribose) polymerase (PARP) and DNA ligase IIIa (LIG3a). In CML, Artemis and LIG4 have been found to be downregulated, whereas LIG3a and the Werner helicase (WRN) are upregulated and form a novel complex acting on DSBs in BCR-ABL1-positive cells [37]. BCR-ABL1 has later been found to induce WRN mRNA and protein expression by c-MYC-dependent activation of transcription and BCL-Xl-dependent inhibition of caspase cleavage, respectively [38]. BCR-ABL1 can complex with and phosphorylate WRN, stimulating its helicase and hexonuclease activites [38] increasing the infidelity of NHEJ and enhancing the activity of the more unfaithful HR and SSA. SSA mediates the annealing of complementary single strands formed after extensive resection at DSBs until repeated sequences are detected. BCR-ABL1 has been reported to stimulate SSA by upregulating RAD52 and ERCC1, which may also result in an accumulation of chromosomal aberrations such as translocations, partial deletions (e.g tumor suppressor genes) and duplications.

The mismatch repair (MMR) system has the role to maintain genomic stability by detecting misincorporated nucleotides, by acting an excision repair before point mutations emerge and by inducing cell apoptosis in case of unrepairable DNA damage. Following the observation that a certain degree of microsatellite instability can be observed at multiple loci in BP-CML differently to CP-CML [39], Stoklosa et al. have demonstrated that the BCR-ABL1 kinase is responsible for reduced MMR activity. The efficacy of MMR was reduced ∼2-fold in BCR-ABL1-positive cell lines and in CD34+ CML cells as compared to normal counterparts. Impaired MMR activity in leukemia cells was associated with accumulation of p53 but not of p73, and lack of activation of caspase 3 after treatment with DNA damage-inducing agents. This resulted in a 15-fold increase in mutation rate in BCR-ABL1-positive cells. The resulting ‘’mutator phenotype’’ may be the primary responsible for the accumulation of point mutations in the BCR-ABL1 kinase domain (KD) sequence and in other key genes, causing TKI-resistance, and promoting the disease progression. Defective base excision repair (BER) may also contribute to the accumulation of point mutations. BER is primarily responsible for the correction of DNA damage by oxidation, alkylation, and deamination processes. In the stem/progenitor cell compartment, BCR-ABL1 has been found to inhibit in a kinase-dependent manner the activity of uracil-N-glycosylase 2 (UNG2), one of the key enzymes that initiates BER by recognizing and eliminating the damaged DNA bases [40].

## 4. Additional Chromosomal Abnormalities

In Philadelphia-positive clones, the classical t(9;22)(q34;q11) can be found in combination with secondary chromosomal aberrations known as additional chromosomal abnormalities (ACAs) [41]. The presence of ACAs can be observed at diagnosis in ≈5% or less of CP CML patients as demonstrated by several studies [42,43,44]. The European LeukemiaNet recommendations suggest that the presence of ACAs at diagnosis should be considered a “warning” sign for patients in early CP, requiring a closer monitoring of this subset of patients. The appearance of ACAs on treatment is well known as clonal evolution (CE) and it has been associated with disease evolution since ACAs frequency is higher in advanced phases, AP (≈30%) and BP (≈80%) [45,46,47]. However, ACAs constitute a heterogeneous collection of karyotype abnormalities with different frequency and different prognostic significance. Mitelman classified them into “major” and “minor” route chromosomal abnormalities according to the frequency of the pathways of cytogenetic evolution followed by CML cells during disease progression. The most common ACAs involved in karyotype evolution are trisomy 8 (the most frequent), a second Ph chromosome, an isochromosome 17 (i(17q)) and trisomy 19. These aberrations are called “major route changes” [48,49]. At least one of these four major karyotipic changes is found in 71% of CML cases with cytogenetic abnormalities in addition to the Ph chromosome [49]. Some among the infrequent cytogenetic changes are trisomies of chromosomes 17 and 21, monosomies of chromosomes 7, 17 and 21, translocations t(3;12), t(4;6), t(2;16), and t(1;21). They are known as “minor route changes” and at least one of them occurs in 15% of all Ph-positive CML patients with additional cytogenetic abnormalities [49]. The lack of the Y chromosome, observed in 5% of Ph+ patients [49], has also been recognized as a minor route ACA. The prognostic significance of ACAs in CP CML patients at diagnosis is still debated. Whereas some studies associated the presence of ACAs in general [50,51,52] or major route abnormalities to worse progression-free survival and/or overall survival [53,54,55] others failed to find any significantly association [8,56].

## 5. Gene Mutations and Submicroscopic Genetic Abnormalities

The most frequently mutated gene in CML is *BCR-ABL1* itself. Point mutations within the *ABL1* KD may indeed be selected during TKI therapy, leading to treatment failure. Mutations disrupt critical contact residues between the TKIs and their target, or induce a shift to a conformation that TKIs may be unable to bind (e.g, from the closed to the open conformation of the kinase, that imatinib and nilotinib cannot recognize). The frequency of BCR-ABL1 KD mutations [57] is relatively low (25% to 30%) in CP patients on first-line therapy but increases up to 70%–80% in BP patients [58,59]. BP patients, much more frequently than CP patients, may accumulate multiple BCR-ABL1 KD mutations as a result of sequential therapy with TKIs. Multiple mutations arising in the same molecule (compound mutations) can be observed in more than 30% of BP patients and are particularly challenging [9,60,61,62,63]. In vitro studies [64] have shown that they are highly resistant to 2GTKIs and in some cases even to ponatinib. Beyond BCR-ABL1 KD point mutations, other genetic lesions have been described with different patterns in myeloblastic and lymphoblastic transformations. The most common detectable gene alterations ‘’historically’’ reported in myeloid BP cases (20% to 30%) before the advent of high throughput technologies, involved the tumor suppressor gene TP53, mapping at 17p13.1 [65]. These alterations usually consist in the loss of one TP53 allele (e.g., as a consequence of structural alterations like i(17p)) and in point mutations in the coding sequence of the remaining allele [66].The p53 tumor suppressor is the master regulator of the DNA damage checkpoint. Mice injected with p53-deficient BCR-ABL1-expressing bone marrow cells developed a more aggressive disease than those injected with the wild-type p53 counterpart [67]. Blastic transformation was also observed in a transgenic model in which mice expressing BCR-ABL1 under the control of the Tec promoter were crossed with p53-heterozygous (p53+/-) mice [68]. Functional p53 has been involved in imatinib-induced apoptosis [69]. *RB1* inactivating mutations or deletions are the second most frequent genetic abnormality detected in myeloid BP (20% of cases) [70,71].The RB tumor suppressor protein restricts cell cycle progression at the G1/S checkpoint until a cell is ready to divide. Point mutations within the DNA-binding region of the *RUNX1* gene were first described in association with trisomy 21 in some myeloid BP [72]. A later study in a large cohort of Chinese CML-AP and CML-BP patients identified missense, nonsense, and frameshift *RUNX1* mutations displaying a reduced transactivation activity and/or a dominant-negative function over the wild-type *RUNX1* in 12% of the cases [73]. The *RUNX1* gene, located on chromosome 21q22, encodes the major subunit of the heterodimeric core binding factor (CBF) complex, a transcription factor essential for myeloid differentiation. In a mouse model, *runx1* mutations were found to perturb myeloid differentiation and induce an AP or BP–like phenotype [73]. Chromosomal translocations involving the *RUNX1* gene have also been reported in BP-CML. The t(3;21)(q26;q22) translocation can be observed in BP CML patients and also in therapy-related AML and myelodysplasia (MDS) [74,75,76,77,78]. Consequently to this translocation, *RUNX1* resulted to be fused with *EAP*, *EVI1* and *MDS,* or both *MDS1* and *EVI1*. *EVI1* (also known as *MECOM* or *PRDM3*) plays an important role as regulator of self-renewal in hematopoietic stem cells [79]. A study in a transgenic zebrafish model showed the capability of this fusion gene to inhibit myeloid cell differentiation and to induce resistance to apoptosis [80].

In lymphoid BP, one of the most frequent abnormalities (detected in approximately 50% of cases), known even before the advent of genome-wide technologies [57,81,82] is represented by monoallelic or biallelic deletions of the *CDKN2A/*B locus, encoding the tumor suppressors and cell cycle regulators INK4A, INK4B, and ARF. The *CDKN2A* and *CDKN2B* genes encode p16^INK4A^ and p15^INK4B^, which are inhibitors of cyclin-dependent kinases. In addition, transcription of an alternate reading frame of the *CDKN2A* locus produces p14^ARF^, which antagonizes the p53 ubiquitin ligase, MDM2. Thus, the INK4-ARF locus regulates both RB and p53 function to prevent an inappropriate progenitor cell self-renewal and to eliminate cells driven by oncogenic signaling [83].

The recent advent of high throughput technologies like DNA microarrays and next generation sequencing (NGS) has contributed to further unravel the genetic/genomic complexity of BP cells. Array-CGH and SNP-arrays [22,23,24,25,26,84,85]; have highlighted the presence of multiple recurrent submicroscopic genomic lesions in myeloid and lymphoid BP. In the latter, as well as in acute lymphoblastic leukemias, such methodologies have enabled the characterization of IKZF1 loss [86]. The *IKZF1* gene on chromosome 7 encodes IKAROS, a transcription factor essential for lymphoid development. Detected early in hematopoietic development, IKAROS expression plays a key role in shutting down stem-cell programs and driving cells towards lymphoid development [87,88]. Monoallelic and bi-allelic deletions of the whole gene or focal in-frame deletions of exons 4 to 7, creating a dominant negative isoform (Ik6) lacking the DNA-binding domain have been reported [86,89,90,91].

NGS has also contributed to pinpoint sequence and structural alterations associated with TKI-resistance and disease progression. An IDH2 mutation was detected by whole transcriptome sequencing in a CML case who had progressed to lymphoid BP while on nilotinib treatment and subsequently reported in 3/75 myeloid BP and 1/31 lymphoid BP patients [92]. No IDH1 mutation emerged in that study. Using a targeted NGS approach to screen for mutations 12 candidate genes in 39 BP cases, Grossman et al. reported mutations in *RUNX1, IKZF1, ASXL1, WT1, TET2, CBL, IDH1, NRAS, KRAS,* and *TP53* overall occurring in 76.9% of the patients. *RUNX1, ASXL1* and *IKZF1* had the highest mutation rate [93]. Makishima et al. screened Janus kinase *(JAK)-2, CBL, CBLB, TET2, ASXL1, IDH1,* and *IDH2* coding sequences in 26 BP patients, identifying four *TET2*, two *ASXL1*, two *IDH1/2*, one *CBL* and one *CBLB* mutations in myeloid BP [94]. A whole exome sequencing analysis of 10 matched CP-BP samples has more recently uncovered that *UBE2A* mutations may recurrently (16.7% of cases in the validation cohort) be acquired at the time of progression [95]. Functional studies have revealed that mutations abrogate UBE2A activity, leading to an impairment of myeloid differentiation [95]. At present, the largest study taking advantage of an integrated approach of whole-exome sequencing, copy-number variation (CNV) analysis and RNA sequencing in CML patients has been performed by Branford et al. [96]. This study has revealed one or multiple mutations in all BP patients tested, CNVs in 90% of cases and gene fusions other than *BCR-ABL1* in 42% of cases [96]. Genes affected by missense, nonsense, frameshift, or splice site mutations included *BCR-ABL1, ASXL1, RUNX1, IKZF1, BCOR, BCORL1, GATA2, PHF6, SETD1B, SETD2, U2AF1, IDH1/2, KMT2D,* and *XPO1*. Among CNVs, a novel recurrent 60-Kb deletion comprising exons 1 to 2 of the *HBS1L* gene and an intergenic sequence between *HBS1L* and *MYB* was detected in 3 of 10 patients with lymphoid BP. Known and novel fusions were also detected by RNA-seq. *MLL* (*KMT2A*) fusions (some of whom were cytogenetically cryptic) were the most frequent. The other known fusions were *CBFB-MYH11* and *PAX5-ZCCHC7*. Novel fusions resulting from intra-or inter-chromosomal rearrangements and mostly cytogenetically cryptic involved cancer genes implicated in hematologic malignancy: *RUNX1, IKZF1, MECOM*, and *MSI2*. They included *RUNX1-MX1*, *MBNL1-MECOM*, and *MSI2-PRMT2*. When matched CP and BP samples from the same patient could be analyzed, some gene mutations like *ASXL1, IKZF1* and *SETD1B* were found to precede disease progression [96].

## 6. Non Genomic Loss of Tumor-Suppressor Function

In BP-CML, loss of function of key tumor suppressors may also be mediated by events not acting at the genomic level. The best example is represented by protein phosphatase A (PP2A). PP2A is one of the major cellular serine-threonine phosphatases and is directly involved in a multitude of cellular processes by dephosphorylating a wide variety of key target proteins (MYC, BCL-2, MEK/ERK, AKT, RB, JAK2, and β-catenin), hence providing a negative feedback to signals triggers and sustained by protein kinases [97]. As such, it has been shown to negatively regulate G2 to M transition, inhibit mitogenic signals, promote apoptosis, and interfere with WNT signaling. Inactivation of PP2A at the genetic or functional level has been shown to play a crucial role in cancer progression. In CML, PP2A inhibition is accomplished mainly through the formation of an inhibitory complex with the phosphoprotein SET. Neviani et al. have shown that, in CML BP progenitors, SET expression is markedly upregulated by BCR-ABL1 in a dose and kinase-dependent manner via induction of hnRNP A1 [98]. Direct phosphorylation of one of PP2A subunits by BCR-ABL1 itself or by other kinases like JAK2 or SRC may also contribute to the suppression of its phosphatase activity. BCR-ABL1 and PP2A share several targets. Among these, MYC, STAT5, MAPK, AKT, BAD and RB are essential for BCR-ABL1-mediated leukemogenesis and have been found altered in BP. Indeed, in BCR-ABL1-expressing myeloid progenitor 32Dcl3 cells, inhibition of SET expression or forced expression of PP2A decreased MYC expression, increased the levels of proapoptotic BAD and led to inhibition of MAPK, STAT5 and AKT phosphorylation thus inducing growth suppression, enhancing apoptosis and restoring differentiation. Additionally, PP2A-induced activation of the SHP-1 tyrosine phosphatase has been shown to promote BCR-ABL1 tyrosine dephosphorylation (inactivation) which, in turn, triggers its proteasome-dependent degradation in CML BP progenitor patient cells [98].

## 7. Differentiation Arrest

In CML, the transition from CP to BP is characterized by a progressively increasing impairment of myeloid blast differentiation which determines the stack of myeloid progenitors. Both BCR-ABL1-dependent and BCR-ABL1-independent mechanisms interfering with differentiation pathways have been described. For example, the marked downregulation of the lineage-specific transcriptor factor CCAAT-enhancer binding protein alpha (C/EBPα) induced by high BCR-ABL1 levels plays a crucial role in the arrest of myeloid cell development [99]. Indeed, C/EBPα expression rises with the commitment of multipotential precursors to myeloid differentiation: its upregulation drives the granulopoiesis while its downregulation contributes to monopoiesis [100]. As a result of genetic and epigenetic alterations, C/EBPα activity may be repressed at different levels in myeloid leukemias. As matter of fact, the increase in BCR-ABL1 expression, marking the transition from CP to BP, is associated with suppression of C/EBPα expression at the translational level. Perrotti et al. showed that in myeloid precursor 32Dcl3 cells transfected with BCR-ABL1 and in primary bone marrow cells from individuals with CML-BP, suppression of C/EBPα protein expression is due to the interaction between CEBPA mRNA with the RNA-binding protein hnRNP E2 [99]. hnRNP E2 and C/EBPα expressions resulted to be inversely correlated: the first one was undetectable in normal marrow and in CML-CP mononuclear cells, which regularly express C/EBPα. Conversely, hnRNP E2 expression was high in CML-BP samples lacking C/EBPα [99]. hnRNP-E2 is post-translationally induced by BCR-ABL1 in a dose and kinase-dependent manner through constitutive activation. ERK1/2 phosphorylate hnRNP-E2, increasing protein stability [101,102]. Following the observation of an inverse correlation between miR-328 and hnRNP E2 expression and between miR-328 and BCR-ABL1 expression and activity in CML-BP progenitors, it was later found that the BCR-ABL1/MAPK/hnRNP-E2/C/EBPα network also involves miR-328 [103]. miR-328 was found to compete with C/EBPα mRNA for binding to hnRNP-E2, thus interfering with hnRNP-E2-mediated inhibition of C/EBPα translation, and to silence the expression of the pro-survival PIM1 kinase through the interaction with its 3′-UTR. Additionally, C/EBPα was found to bind miR-328 promoter, thus enhancing its transcription. It was thus demonstrated that BCR-ABL1 uses the MAPK/hnRNP-E2/C/EBPα pathway to suppress both C/EBPα protein expression and miR-328 transcription [103]. Additional mechanisms acting at the genetic level may contribute to the arrest of myeloid or lymphoid development. As detailed in the chapters above, many recurrent mutations and copy number alterations both in myeloid and in lymphoid BP involve transcription factors regulating myeloid (GATA2, UBE2A, RUNX1) or lymphoid (CDKN2A, Ikaros) differentiation.

## 8. Methylation Changes

Epigenetic changes are known to cooperate with genetic events in human tumorigenesis. Although aberrant hypermethylation in CML had previously been described [104,105,106,107,108,109], the role of altered DNA methylation in CML progression is still to be deeply investigated. There is a strong evidence that methylation of CpG islands (CGIs) is associated with transcriptional gene silencing. In this regard, Jelinek et al. studied the methylation status of promoter-associated CpG island of 10 genes (*ABL1, CDH13, DPYS, CDKN2B, OSCIP1, PGR-A, PGR-B, TFAP2E, NPM2, PDLIM4)* in CML patients observing a remarkable methylation increase underlying the progression from AP to BP [110]. A correlation between an increased methylation status of these genes and resistance or intolerance to imatinib was also found [110]. Hypermethylation of the tumor suppressor *PDLIM4* was found to be a negative prognostic factor independent from imatinib response and from CML phase [110]. Thank to the high throughput of NGS, Heller et al. [111] investigated the methylome of CML patients in CP, AP, and BP as compared to controls. CpG site methylation was found to increase dramatically (up to 58,691 differentially methylated CpG sites) during progression from CP to AP/BP. Interestingly, they were able to localize the CpG sites associated with a higher methylation status in the advanced CML stages in or around CGIs, but also in the binding sites of specific transcription factors. Taking advantage of RNA-seq, they were also able to identify the genes that are transcriptionally regulated by methylation in BP samples. Some of these genes were known to be implicated in the pathogenesis of various malignancies, but their exact role in CML progression remains to be further investigated.

## 9. Perturbations in RNA Transcription, Editing and Translation

The increased BCR-ABL1 expression observed in BP CML has also been linked to an aberrant regulation of processing, nuclear export and translation of mRNA. Aberrant expression of various RNA-binding proteins (RBPs) with post-transcriptional and translational regulatory roles has been observed in mouse models and primary blasts from CML patients. RNA-binding proteins regulate transcription, maturation, nucleocytoplasmic transport, RNA stability and translation; some of them are general regulators while others recognize given mRNAs in a sequence-specific manner. Besides the already mentioned interplays between hnRNP E2 and C/EBPα, and between hnRNP-A1 and SET/PP2A, other RBPs that have been implicated in CML progression are: HNRPK, which binds the internal ribosome entry site (IRES) element of MYC mRNA and enhances its translation in a BCR-ABL1 and MAPK-dependent manner [101]; La/SSB, which enhances MDM2 translation contributing to p53 inactivation [112]; FUS/TLS, which binds the granulocyte colony-stimulating factor receptor (G-CSFR) pre-mRNA in the nucleus and interferes with its processing and export [113]; and CUGBP1, whose loss results in a reduced translation of C/EBPβ [114]. hnRNP-A1 has also been found to bind the mRNA encoding the E2F3 transcription factor [115] leading to its upregulation in BP CML progenitors [116,117]. Different BCR-ABL1-dependent mechanisms underlie the aberrant expression and/or function of RBPs, some of whom involve phosphorylation events by PI-3K/AKT, ERK or PKC. Overall, these phenomena contribute to differentiation arrest and resistance to apoptosis of BP-CML progenitors by either the loss of function of tumor suppressors, upregulation of oncogenes, or altered expression of regulatory factors essential in differentiation processes.

There is also evidence that BCR-ABL1 interferes with the efficiency of the basal machinery responsible for mRNA translation. The latter is accomplished via regulation of the mTOR and S6 kinase pathway and of the EIF4E e EIF4E-BP translation factors [116,117].

More recently, a massively parallel RNA sequencing approach has revealed an increased expression of the adenosine deaminase acting on dsRNA-1 (ADAR-1) in progenitor cells obtained from primary BP CML patient samples. ADAR-1 performs adenosine to inosine (A to I) RNA editing of double strand RNA hairpins formed between Alu repetitive elements, thus altering mRNA structure, generating or abolishing donor and acceptor splice sites and introducing sequence alterations. Functional studies demonstrated that ADAR-1-mediated RNA editing plays a role in the malignant reprogramming of myeloid progenitors into LSC that drive CML progression, by promoting the expression of the myeloid transcription factor PU.1 and producing misspliced isoforms of GSK3β [118]. The increased expression and activity of ADAR-1 was found to be mediated by inflammatory cytokine-activated JAK/STAT signaling as well as by BCR-ABL1 signaling [119]. Other ADAR-1 targets were found to be MDM2, APOBEC3D, GLI1 and AZIN [120] and the miRNA Let-7 [119]. In particular, ADAR-1 hyper-editing of the 3′-UTR of MDM2 prevents miRNA binding, thus resulting in increased MDM2 expression and repression of the p53 tumor suppressor [118].

## 10. The Role of MicroRNAs

MicroRNAs (miRNAs) are known to play an essential role in tumorigenesis [121,122,123] by post-transcriptional regulation of gene expression. Aberrant expression of several miRNAs has been described in CML, in association with stem cell survival and self-renewal, sensitivity or resistance to TKI therapy, and disease progression. In the latter context, a microarray analysis revealed differential expression profiles of several miRNAs–namely, the up-regulation of miR-19a, miR-19b, miR-17, miR-20a, miR-92a, miR-221, miR-222, miR-126, miR-146a, miR-181a, miR-181b, let7c, and miR-155 and the down-regulation of miR-150, miR-452, miR-103, and miR-144–in BP samples [124]. The association between reduced miR-150 level, myeloid differentiation block and resistance to TKIs was later found to be due to a novel mechanism whereby BCR-ABL1 recruits MYC to bind and inhibit miR-150 expression [125].

As previously mentioned, another key miRNA is miR-328, that is involved in C/EBPα downregulation and in the subsequent block of myeloid differentiation [99,103].

## 11. Metabolic Changes

All cancer cells develop an altered metabolism to support their growth and survival. Recently, it has been suggested that increased branched chain amino acid (BCAAs; valine, leucine, and isoleucine) metabolism may contribute to disease progression in CML. Following the observation that mice with BP CML display higher levels of proline and BCAAs, Hattori et al. found a significant upregulation of branched chain amino acid aminotransferase 1 (*BCAT1)* at the mRNA and protein levels in BP-CML as compared to CP-CML or healthy mice [126]. Similarly, patients with BP-CML showed higher *BCAT1* expression as compared to CP CML patients. *BCAT1* is a cytosolic aminotransferase that may generate BCAAs from branched chain ketoacids. BCAAs, particularly leucine, activate the mTORC1 pathway, which promotes cell growth as a result of the integration of nutrient sensing, energy status, stress and growth factors [127]. *BCAT1* upregulation was found to result from association with Musashi 2 (Msi2), an oncogenic RNA binding protein that post-transcriptionally regulates gene expression and that was already known to be upregulated and to cooperate in disease progression via repression of the cell fate regulator Numb [128,129].

## 12. TKI Resistance and LSC Persistence: Two Sides of the Same Coin?

Three levels of response to therapy are routinely defined in CML: hematological response, cytogenetic response (reduction in the percentage of BM metaphases positive for the Philadelphia chromosome) and molecular response (reduction in BCR-ABL1 transcript levels, measured in terms of logarithmic reduction from a standardized baseline) [30,130]. Nowadays, the term ‘’resistance’’ is used to label a wide and heterogeneous spectrum of ‘’non optimal’’ levels of response to TKI therapy ranging from failure to achieve a major molecular response (MMR; defined as BCR-ABL1 transcript levels at or below 0.1%) or increase in BCR-ABL1 transcript levels leading to a loss of MMR, to a frank loss of hematological response. This is essentially due to the fact that patients’ and physicians’ expectations regarding treatment endpoints have increased greatly since the advent of TKIs. If, in the early days of the imatinib era, the endpoints were still ‘limited’ to improving the overall survival and delaying disease progression, it was soon realized that molecular responses could be achieved in a significant proportion of patients and correlated with superior outcomes (hence the definition of MMR as ‘’safe haven’’) [131]. More recently, the endpoint has become even more ambitious―deep molecular response (DMR; defined as BCR-ABL1 transcript levels at or below 0.01%), because it correlates with optimal long term survival outcomes and because it is the pre-requisite for treatment discontinuation. The fact that very low levels of residual disease may still be detected after many years of therapy in a not negligible proportion of patients, and that even when molecularly undetectable the disease may often re-emerge after TKI discontinuation is referred to as ‘’persistence’’. The mechanisms responsible for resistance are likely to be at least in part different from those underlying disease persistence at the molecular level. Resistance mechanisms have extensively been reviewed elsewhere [132,133,134]. In an attempt to fill the gaps in knowledge about BCR-ABL1-independent mechanisms that may enable Ph+ cells to survive despite TKI-mediated BCR-ABL1 inhibition, recent research studies have highlighted a number of pathways/molecules that might also be therapeutically targeted (summarized in Table 1).

**Figure 3:**
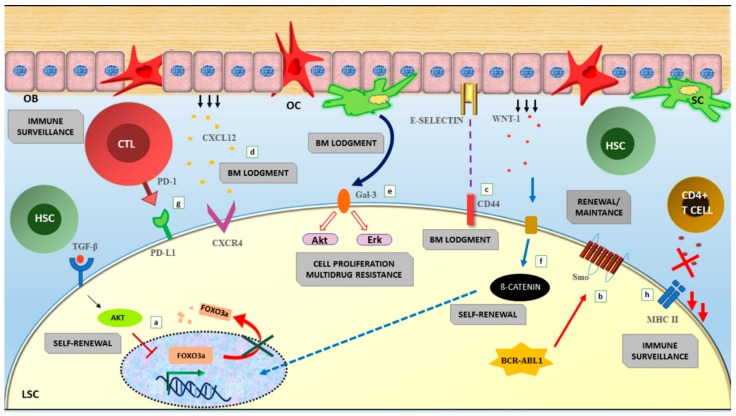
Persistence of LSCs: BCR-ABL1 kinase-dependent and -independent pathways.

Persistence is thought to be mainly attributable to the fact that TKIs may eliminate differentiated and progenitor cells, but spare LSCs. BCR-ABL1-positive primitive cells are indeed detectable in the BM in patients with MMR and even DMR [150,151,152,153,154]. The fact that LSCs are intrinsically insensitive to TKIs was observed early after the introduction of first and second-generation TKIs [150,155,156,157]. Later on, a series of experiments in cell lines and mouse models suggested that LSC are not dependent from BCR-ABL1 kinase activity for their survival, and that BCR-ABL1 may possess non kinase-mediated functions influencing signaling pathways responsible for LSC survival [157,158,159,160]. It has also been hypothesized that while BCR-ABL1 expression is high in pre-therapy CD34+ cells, TKIs select for CML precursors with low BCR-ABL1 expression and signaling, hence less oncogene-addicted [161,162]. This has sparked searches for LSC-selective BCR-ABL1 kinase-independent pathways. To date, a number of cell- intrinsic and cell extrinsic pathways and mechanisms have been suggested to contribute to the TKI-resistant LSC phenotype, and many of them are potentially druggable. Among the cell-intrinsic mechanisms, Foxo [163,164], Sonic Hedgehog [165,166] and Wnt/β-catenin [167,168] pathways are the most extensively investigated ones. However, interaction with the BM microenvironment is thought to be equally critical for LSC survival. CD44/E-selectin [169,170], galectin-3 (Gal-3) [171], CXCR4/CXCL12 [172] have all been suggested to enhance LSC self-renewal. Last but not least, it has been hypothesized that modulation of host immune surveillance in the BM microenvironment may have a role in preventing LSC eradication. In this regard, cytotoxic T lymphocytes exhaustion via interaction of the PD-1 receptor expressed on cytotoxic T lymphocytes with its inhibitory ligand PD-L1 expressed on CML cells has been observed [173,174]. Cytokine-mediated downregulation of Major Histocompatibility Complex class II (MHC-II) molecule expression has been reported to be an alternative way for LSCs to evade immune surveillance [175]. All these mechanisms are summarized in Figure 3.

Schematic representation of the key pathways and molecules known to be implicated in LSC persistence in CML. **a**) Foxo3a plays a key role in the TGF-β signalling pathway driving the survival of leukemia initiating cells (LICs) during TKI treatment. In LICs, despite BCR-ABL1 expression in all CML cells, Akt is inactivated and as consequence, the Foxo proteins are retained in an active form in the nucleus inducing transcription and maintenance of LSCs. TGF-β signalling is activated in CML LICs, where it controls Foxo localization.

**b**) In the Sonic/Hedgehog pathway, Smo is upregulated in BCR-ABL1-positive LSCs. On the other hand, it is not necessary for the long-term regeneration of LSCs. **c**) BCR-ABL1-positive LSCs are more dependent from an higher expression of CD44 for homing and engraftment as compared to the HSCs. **d**) Reduced CXCL12 expression in BM stromal cells determines an impaired LSC homing and BM retention. **e**) High levels of Gal-3 expression in BM support disease maintanence, multidrug resistance to TKIs and long-term BM lodgment of CML cells. **f**) Expression of β-catenin in LSCs determines an increased growth and a reduced differentiation. **g**) The interaction between the PD-1 receptor expressed on CTLs and the inhibitory ligand PD-L1 present on CML cells enables the latter to escape from immune surveillance. **h**) The antitumor-associated immune response exercised by CD4+ T helper cells is prevented by the downregulation of the MHCII. Abbrevations: LSC (leukemia stem cell), OB (osteoblast), OC (osteoclast), SC (staminal cell), HSC (hematopoietic stem cell), CTL (cytotoxic T lymphocyte), LIC (leukemia initiating cell).

## 13. Conclusions and Future Perspectives

Though rare, resistance to TKI therapy and disease progression remain a concern for physicians and patients. Whenever resistance occurs, reactivation of BCR-ABL1 kinase activity represents a ‘‘fire under the ashes’’ that may ultimately lead to the evolution from CP to BP―which represents a clinical emergence even in the era of TKIs. The lack of a unifying path of disease progression and the profound molecular heterogeneity of BP CML patients limit the effectiveness of therapeutic options once BP is diagnosed―which strongly underline the importance of preventing rather than treating progression. High risk patients may be identified using scores like Sokal or EUTOS [3]. Major route ACAs may also help identify patients at greater risk of progression [53]. Some biological factors have also been proposed in single studies (e.g., germline variants in *ASXL1* and *BIM* genes [176,177]; CIP2A protein levels at diagnosis [178]). So far, however, no validated prediction algorithm has ever been derived that may soundly predict who will develop BP. Thus, strict MR monitoring and thoughtful TKI therapy reassessment in case of failure to achieve the key response milestones are currently the only way to minimize the risk of progression.

Even in patients achieving deep molecular responses to TKI therapy, LSCs have been reported to persist, constituting a dangerous reservoir that may feed disease recurrence in case of treatment discontinuation. Twenty years of research have resulted in a wealth of biological acquisitions. However, very few clinical trials evaluating the combination of TKIs with agents aimed at selectively targeting LSC are underway [179]. Hence it is currently difficult to predict whether a successful and well-tolerated eradication strategy will ever be defined.

## Figures and Tables

**Table 1:** Overview of the molecules and pathways that have been implicated in BCR-ABL1-independent resistance.

Gene/Pathway	Ref	Druggable?
PI3K/AKT/mTOR	Burchert, Leukemia 2005 [135]	PI3K/mTOR inhibitors
Lyn	Wu et al., Blood 2008 [136]	Src inhibitors
Autophagy	Bellodi et al., J Clin Invest 2009 [137]	hydroxychloroquine
*SHP-1*	Esposito et al., Blood 2011 [138]	X
*SIRT1*	Wang et al., Oncogene 2013 [139]	selisistat
*PRKCH*	Ma et al., Sci Transl Med 2014 [140]	MEK inh (trametinib) + Imatinib
*STAT3*	Eiring et al., Leukemia 2015 [141]	BP-5-087
*CRM1/XPO1/RAN*	Khorashad et al., Blood 2015 [142] Walker et al., Blood 2016 [143]	selinexor
*JAK2*	Chakraborty et al., Genes Cancer 2016 [144]	ruxolitinib
*FOXO1*	Wagle et al., Leukemia 2016 [145]	PI3K inhibitors
*EZH2*	Scott et al., Cancer Discov 2016 [146]	*EZH2* inhibitors
Wnt/b catenin	Eiring et al., Leukemia 2015 [141], Zhou et al., Leukemia 2017 [147]	C82
*MS4A3*	Eiring et al., ASH 2017 [148]	X
*PFKFB3*	Zhu et al., Oncogene 2018 [149]	PFK-158
Various miRNAs		X
